# Efficient Removal of As from Industrial Wastewater by Nanocomposite MnFe_2_O_4_@Fe-UiO-67

**DOI:** 10.3390/toxics13040295

**Published:** 2025-04-11

**Authors:** Mengmeng Geng, Xianjin Qi, Junwei Feng

**Affiliations:** State Key Laboratory of Complex Nonferrous Metal Resources Clean Utilization, Faculty of Metallurgical and Energy Engineering, Kunming University of Science and Technology, Kunming 650093, China

**Keywords:** MnFe_2_O_4_@Fe-UiO-67, efficient adsorption, arsenic, monomolecular layer chemisorption

## Abstract

Arsenic is a highly toxic element, and excessive levels can affect human health. Composites possess a larger specific surface area and better adsorption performance than single-MOF materials. In this paper, a simple novel nanocomposite (MnFe_2_O_4_@Fe-UiO-67) was synthesized by the one-pot method for the removal of arsenic from industrial wastewater. The synthesis and adsorption mechanism of the adsorbent were analyzed by a series of characterizations. The results showed that the adsorption behavior of MnFe_2_O_4_@Fe-UiO-67 was consistent with the pseudo-secondary kinetics and Langmuir isotherm model, i.e., it is a monomolecular layer chemisorption. Characterization by Fourier transform infrared (FT-IR) and X-ray photoelectron spectroscopy (XPS) showed that the active site formed a strong coordination bond (As-O bond) with As ions to achieve efficient adsorption. At 298 K and pH = 10, the arsenic removal rate can reach 98.43%, and the adsorption capacity is 600.25 mg/g, which is more than most of the existing reported adsorbents. Through thermodynamic analysis, it is found that the adsorption of As ions by the adsorbent is a spontaneous exothermic process. It can exhibit excellent adsorption performance at room temperature without the need for additional energy consumption. This adsorbent has great development prospects in the treatment of wastewater.

## 1. Introduction

Arsenic exists in nature mainly in the form of trivalent and pentavalent compounds; trivalent compounds are more toxic than pentavalent compounds [1,2]. Arsenic mainly comes from the smelting of non-ferrous metals, the mining of arsenic-containing minerals, and the application of arsenic-containing compounds in industrial production, such as paint, glass, textile, semiconductor, and nitrogen fertilizer [3,4]. Arsenic compounds often exist in the form of by-products, and in the wastewater discharged into the environment, causing harm to the human body, such as neurasthenia, cardiovascular disease, diabetes and so on [5]. Therefore, the World Health Organization (WHO) recommends a maximum concentration limit of 10 μg/L for drinking water [6].

Currently, arsenic removal methods include chemical precipitation [7], adsorption [8], and microbial methods [9]. Each method has its own advantages and disadvantages, but currently, adsorption is considered the most promising method because of its high efficiency, suitability for industrial application, low cost, simple operation, and high regeneration capacity [10,11]. In the past, a variety of adsorbents have been developed and applied for the treatment of arsenic-containing wastewater. For example, Ahmad Sadeghi Chevinli et al. [12] synthesized a Mg-Fe LDH-GO adsorbent with high adsorption capacity for both As (III) and As (V) by the co-precipitation method. Xiaoli Song et al. [13] loaded Fe_3_O_4_ on halloysite nanotubes (HNTs), and experiments have shown that the nanocomposites have highly efficient arsenic removal performance and recyclability. Conventional adsorbents such as activated carbon, clay, zeolite, and agricultural wastes exhibit limited arsenic removal efficiency, and thus new adsorbent materials have been developed for the removal of arsenic from industrial wastewater [14,15].

Metal–organic frameworks (MOFs) are composed of metal ions or clusters coordinated with organic linkers, which exhibit an exceptional affinity toward metal ions, and are used as a new type of nanomaterial due to their high specific surface area, large pore size, and good stability [16,17,18]. MOF materials can be used for the removal of various pollutants from water, as shown by Hongfei Ma et al. [19], who investigated the synthesis of La-MOFs in different morphologies by altering the solvothermal temperature and applied them for the removal of fluoride ions (F^−^) from water. Hossein Molavi et al. [20] prepared a series of Ce-MOF materials by using different solvents and thoroughly investigated the effect of such materials on the organic dyes’ adsorption properties. However, single MOFs have fewer applications in the practical treatment of wastewater due to their poor selectivity and weak capture ability. Modifying MOFs is an inevitable trend aimed at increasing the number of adsorption active sites and enable a wider range of applications. The current methods of MOF modification include the doping of metals [21], changing the organic ligands [22], the generation of induced defects [23], the introduction of functional groups [24], etc. In addition, two different forms of MOF are mixed together to obtain new materials with different structures and properties [25].

Spinel ferrites (MnFe_2_O_4_) possess excellent chemical stability and abundant surface functional groups, which are advantageous for removing various toxic pollutants from water [26]. Ting Pan et al. [27] synthesized MnFe_2_O_4_@S-ZnO and investigated its performance in removing norfloxacin (NOF). Tao Zhang et al. [28] successfully prepared a composite material, MnFe_2_O_4_/rGO, through defect engineering, which demonstrated efficient electrochemical arsenic removal performance along with excellent stability. Rauf Foroutan et al. [29] modified biochar (BCSO) to synthesize BCSO/MnFe_2_O_4_@La-MOF for the highly efficient removal of fluoride ions (F^−^) from water. With the increasing awareness of environmental protection, the development of green and eco-friendly materials has become a hot research topic. For example, Sonia Jemli et al. [30] used sunflower seed husk (SFSH) and β-cyclodextrin (β-CD) to green-synthesize a crosslinked adsorbent (SFSH-β-CD) for the removal of phenol and cyclohexane carboxylic acid. Xiang Chen et al. [31] synthesized an eco-friendly adsorbent, 2D/2D Na^+^-MXene/LDH composite, for cesium adsorption in salt lakes.

In this paper, the doping of metals and introduction of spinel ferrite (MnFe_2_O_4_) are carried out to modify MOF materials. Zirconium-based metal–organic frameworks have demonstrated extensive applications due to their high surface area, good stability, and strong adsorption capacity. MnFe_2_O_4_@Fe-UiO-67 composites were used for the adsorption of arsenic from industrial wastewater in this study. The structure and adsorption mechanism of the adsorbent were explored by characterization methods such as X-ray diffraction (XRD), scanning electron microscopy (SEM), Fourier transform infrared (FT-IR), and X-ray photoelectron spectroscopy (XPS), combined with the use of adsorption kinetics and isotherm modeling. In addition, the adsorption performance of the material was explored in terms of pH value and adsorbent dosage.

## 2. Experimental Parts

### 2.1. Experimental Materials

Zirconium chloride (ZrCl_4_, 98%), acetic acid (CH_3_COOH, 99.5%), ferric nitrate nine water (Fe(NO_3_)_3_·9H_2_O, 99.99%), potassium permanganate (KMnO_4_, AR), ferrous sulfate heptahydrate (FeSO_4_·7H_2_O, 99%), N,N-dimethylformamide (DMF, 99%), 4-4′-biphenyldicarboxylic acid (4,4′-H_2_BPDC, 98%), methanol (CH_3_OH, 99.5%) and ethanol (CH_3_CH_2_OH, 75%) were used. All reagents were analytical-grade reagents in this experiment.

The industrial wastewater used in this experiment was from a copper smelting plant and contained Cu, Cd, Ni and other heavy-metal elements; it had a complex composition and high toxicity, as shown in Table 1.

### 2.2. Preparation of Adsorbents

#### 2.2.1. Preparation of MnFe_2_O_4_

MnFe_2_O_4_ was synthesized by dissolving 0.015 mol of KMnO_4_ and 0.045 mol of FeSO_4_·7H_2_O in 200 mL of ultrapure water [32]. The potassium permanganate solution was stirred, and the ferrous sulfate solution was slowly added to the potassium permanganate solution, while 5 M NaOH solution was added to adjust the pH to 10, and after stirring for 3 h, it was static-aged, washed with ultrapure water and dried.

#### 2.2.2. Preparation of MnFe_2_O_4_@Fe-UiO-67

The 4,4′-H_2_BPDC (0.5808 g), ZrCl_4_ (0.2796 g), and acetic acid (2.28 mL) were added to 90 mL of DMF, and stirred until the sample was completely dissolved. Fe(NO_3_)_3_·9H_2_O (0.4848 g) was added and stirred homogeneously, and then 0.1 g of MnFe_2_O_4_ was added and stirred homogeneously. The obtained mixture was transferred to a 200 mL PTFE-lined autoclave and kept at 120 °C for 24 h. After the reaction, the reactor was allowed to cool to room temperature. The solid product was separated by centrifugation, washed with DMF, CH_3_OH, and CH_3_CH_2_OH and dried [33,34].

### 2.3. Methods of Analysis

The crystal structure of the adsorbents was determined using an X-ray diffractometer (XRD, MiniFlex600, Rigaku Corporation, Akishima, Japan) with a scanning range of 0–90° and a scanning speed of 5°/min; a field emission scanning electron microscope (SEM, Apreo 2S, Thermo Fisher Scientific, Waltham, MA, USA) was used to view the microscopic morphology of the adsorbents and surface elements, and the adsorption–resolution isotherm of N_2_ was determined using a Micromeritics ASAP 2460 instrument (Norcross, GA, USA), while the specific surface area of the adsorbent was calculated by the Brunauer–Emmett–Teller (BET) method and the pore volume and pore size distribution were analyzed by the Barrett–Joyner–Halenda (BJH) method. Infrared spectra in the range of 400–4000 cm^−1^ were collected using a Fourier transform infrared spectrometer (FTIR, Thermo Scientific Nicolet iS20, USA) in order to analyze the changes in the chemical bonding of the materials before and after adsorption. X-ray photoelectron spectroscopy (XPS, Thermo Scientific K-Alpha, USA) was used to analyze the elemental composition and content of the adsorbent, and the changes in the valence. An Inductively Coupled Plasma Atomic Emitter (ICP-OES, PQ9000, Jena, Germany) was used to measure the concentration of arsenic ions in solution.

### 2.4. Adsorption Experiments

There are many factors affecting the adsorption performance of adsorbents, and the batch experiments focused on investigating the effects of solution pH, adsorption time, adsorbent dosage, and arsenic solution concentration.

The pH of the arsenic solution was adjusted to 2–12, and then 10 mg of adsorbent was added to 50 mL of the arsenic solution. We then determined the As ion concentration after 24 h of reaction. An amount of 10 mg of the adsorbent was added to 50 mL of the arsenic solution at the optimal pH = 10, and vacuum-filtered through a 0.45 μm membrane the solution at a specific point of time, after which we determined the concentration of As ions. Under optimal reaction conditions (arsenic solution pH = 10, reaction time 3 h), a certain amount of adsorbent (0.1–1 g/L) was added to 50 mL of the As solution to determine the maximum arsenic removal rate. Different concentrations of arsenic solutions (100 mg/L, 200 mg/L, 300 mg/L, 400 mg/L, 500 mg/L, 600 mg/L, 700 mg/L, 800 mg/L, 900 mg/L, 1000 mg/L and 1100 mg/L) were configured to investigate the maximum adsorption amount of MnFe_2_O_4_@Fe-UiO-67 under the conditions of pH = 10 and 3 h of reaction, and the dosage of the adsorbent was 0.8 g/L. In order to systematically explore the adsorption thermodynamic properties of the adsorbent, MnFe_2_O_4_@Fe-UiO-67, this study selected three temperatures (298 K, 308 K, and 318 K) with which to carry out the experiments. Solutions with initial arsenic concentrations of 33 mg/L, 130.5 mg/L, and 210 mg/L were prepared, and the pH value of the solutions was adjusted to 10. Then, 50 mL of the above-mentioned arsenic solution was taken, and 0.8 mg/L of the adsorbent was added to it. The reaction was carried out for 3 h at different temperatures. Finally, the experimental data were collected and their thermodynamic properties are analyzed. In the experiments, to investigate the optimal pH, adsorption time, and adsorbent dosage, arsenic solutions with an initial concentration of 30 mg/L were used. To avoid errors, the above experiments were conducted three times. The As removal rate and adsorption capacity were calculated using Equations (1) and (2):

(1) $$R\left(\%\right)=\frac{C_{0}-C_{e}}{C_{0}}\times 100\%$$

(2) $$q_{e}=\frac{\left(C_{0}-C_{e}\right)V}{m}$$

where *R* (%) is the arsenic removal rate, *C*_0_ (mg/L) is the initial arsenic solution concentration, *C_e_* (mg/L) is the arsenic solution concentration after adsorption, *q_e_* (mg/g) is the adsorbed amount, *V* (L) is the volume of the arsenic solution, and *m* (g) is the mass of the adsorbent, MnFe_2_O_4_@Fe-UiO-67.

### 2.5. Equations for Adsorption Kinetics, Isotherms and Thermodynamic Analysis

Time is an important factor affecting the adsorption performance of an adsorbent. We used the pseudo-first-order model (PFO), pseudo-second-order model (PSO) and intraparticle diffusion model to study the adsorption process [35]. The equations are as follows:

(3) $$\ln\left(q_{e}-q_{t}\right)=\ln q_{e}-k_{1}t$$

(4) $$\frac{t}{q_{t}}=\frac{t}{q_{e}}+\frac{1}{k_{2}q_{e}^{2}}$$

(5) $$q_{t}=k_{3}t^{0.5}+C$$

where *q_e_* (mg/g) is the adsorbed amount at the equilibrium adsorption time, and *q_t_* (mg/g) is the adsorbed amount at a specific time. *k*_1_ (1/min), *k*_2_ (mg/g·min), and *k*_3_ (mg/g·min^0.5^) represent the rate constants for PFO and PSO. *t* is the reaction time (min).

In addition to time, the concentration of the arsenic solution is also an important factor affecting the adsorption performance of MnFe_2_O_4_@Fe-UiO-67. To investigate the adsorption isotherms, Langmuir, Freundlich and Temkin models were used to fit the isotherms to the experimental data [36]. The adsorption model equations are given below:

(6) $$q_{e}=\frac{q_{m}K_{L}C_{e}}{1+K_{L}C_{e}}$$

(7) $$q_{e}=K_{F}C_{e}^{1/n}$$

(8) $$q_{e}=\frac{RT}{B_{T}}\ln\left(K_{T}C_{e}\right)$$

where *q_e_* and *q_m_* are the adsorption amount at the adsorption equilibrium and the maximum adsorption amount of the adsorbent (mg/g), respectively; *K_L_* (L/mg), *K_F_* (L/mg), and *K_T_* (L/g) are the Langmuir adsorption constant, Freundlich’s constant, and Temkin’s constant, respectively; *n* is Freundlich’s model coefficient, and *R* is the gas constant (8.314 J/mol/K).

On the other hand, temperature is a factor that cannot be ignored in the adsorption process. Adsorption thermodynamics -reveals the variations in energy and entropy during the adsorption process, allowing us to make judgments about the direction and extent of the adsorption reaction, and thus enabling a profound understanding of the adsorption mechanism. The relevant thermodynamic formulas are as follows:

(9) $$K=\frac{q_{e}}{C_{e}}$$

(10) $$\Delta G^{0}=-RT\ln K$$

(11) $$\ln K=-\frac{{\Delta H}^{0}}{RT}+\frac{{\Delta S}^{0}}{R}$$

Among them, the Gibbs free energy change (Δ*G*^0^), enthalpy change (Δ*H*^0^), and entropy change (Δ*S*^0^) are the core thermodynamic parameters, with units of kJ/mol, kJ/mol, and kJ/mol/K, respectively; *K* is the adsorption equilibrium constant, *q_e_* (mg/g) is the adsorption capacity, *C_e_* (mg/L) is the remaining arsenic concentration after adsorption, *R* is the gas constant with a value of 8.314 J/mol/K. and *T* (K) is the adsorption temperature.

## 3. Results and Discussion

### 3.1. Characterization of Adsorbents

**Figure 1:**
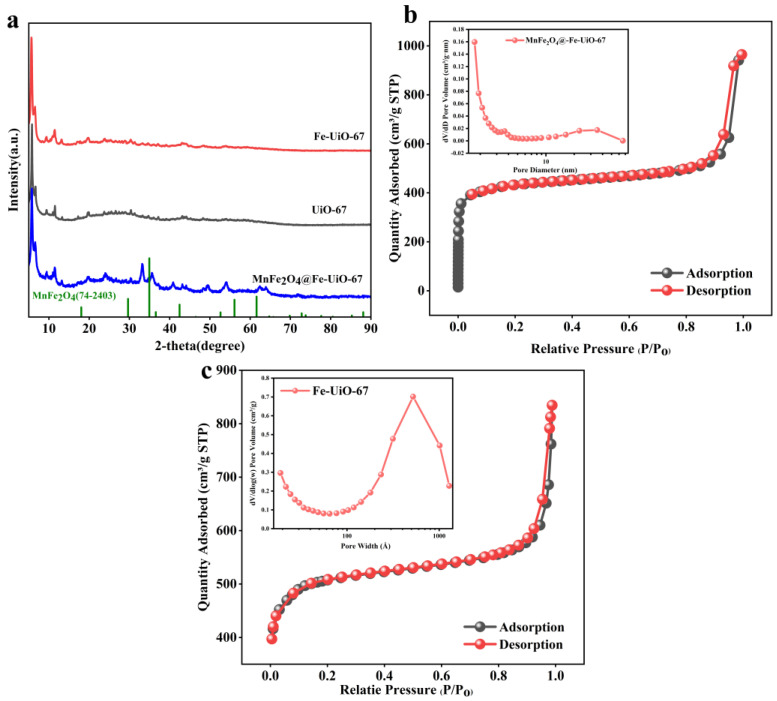
XRD (**a**) and BET (**b**) of MnFe_2_O_4_@Fe-UiO-67, BET of Fe-UiO-67 (**c**).

As shown in Figure 1a, the XRD peaks of UiO-67 and Fe-UiO-67 are the same and are in agreement with those in the literature [37], indicating that there is no change in the structure of UiO-67 caused by the doping of iron ions. The characteristic peaks of MnFe_2_O_4_ in MnFe_2_O_4_@Fe-UiO-67 are shifted when compared with those on the XRD standard card of MnFe_2_O_4_ (JCPDS card no.74-2403). Figure 1a shows that the characteristic peaks of MnFe_2_O_4_ appear at 33.25°, 35.6°, 43.15°, 54.05°, and 62.4° for MnFe_2_O_4_@Fe-UiO-67 [34,38], indicating the successful loading of MnFe_2_O_4_ on Fe-UiO-67. Figure 1b,c show the N_2_ adsorption–desorption curve of the adsorbent, MnFe_2_O_4_@Fe-UiO-67, and its precursor, Fe-UiO-67. According to the IUPAC classification, both show typical type IV isotherms accompanied by type H1 hysteresis loops, indicating that they are mesoporous structures. It is worth noting that although the literature reports the pristine UiO-67 as a microporous material [39], the pore structure of the material is significantly changed by Fe^3+^ doping and composite modification with MnFe_2_O_4_. The surface area, pore size, and pore volume of the adsorbent were calculated by the Barrett–Joyner–Halenda (BJH) method (shown in Table 2). It can be seen that the specific surface area and pore size of MnFe_2_O_4_@Fe-UiO-67 are larger than those of UiO-67 and Fe-UiO-67, resulting in better adsorption performance. However, the pore volume of MnFe_2_O_4_@Fe-UiO-67 is smaller than that of Fe-UiO-67, which may be the reason for partial pore collapse.

**Figure 2:**
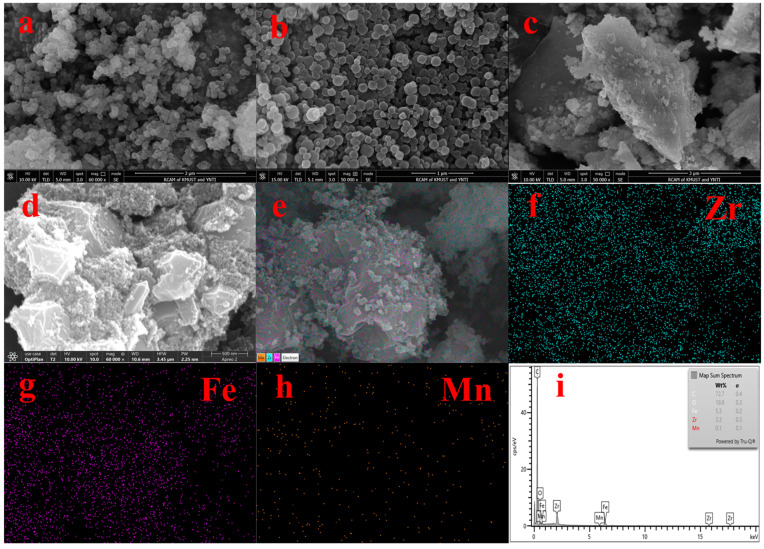
SEM spectra of adsorbents UiO-67 (**a**), Fe-UiO-67 (**b**), MnFe_2_O_4_ (**c**), MnFe_2_O_4_@Fe-UiO-67 (**d**,**e**) and EDS of MnFe_2_O_4_@Fe-UiO-67 (**f**–**i**).

Figure 2 shows the SEM and EDS spectra of the adsorbent. From Figure 2a,b, we can see that UiO-67 and Fe-UiO-67 have the same structure. Figure 2c shows the morphology of MnFe_2_O_4_, which large particles with irregular shape. Figure 2d,e show that the morphology MnFe_2_O_4_ and Fe ions were successfully doped on UiO-67; the rough small particles are Fe-UiO-67 and the large particles are MnFe_2_O_4_ in the SEM image of MnFe_2_O_4_@Fe-UiO-67. The EDS (Figure 2f–i) maps have the elements Zr, Fe and Mn, but the content of Mn is lower, so the EDS map of Mn (Figure 2h) is noisy. The content of Mn in the adsorbent is only 0.86%, as measured by ICP.

### 3.2. Effect of Initial pH on Adsorbent Performance

The form of arsenic ions in solution depends on the pH range. For example, As (V) exists as H_3_AsO_4_ at pH < 2; with increasing pH (2–7), H_3_AsO_4_ ionizes an H^+^ atom to form H_2_AsO_4_^−^. At pH > 7, it exists as HAsO_4_^2−^; at pH > 12, arsenic exists mainly as AsO_4_^3−^ [40]. The surface charge of the adsorbent is also affected by the pH, so we explored the effect of different pH values on the adsorption performance of the adsorbent and analyzed the zeta potential to understand the adsorption mechanism.

**Figure 3:**
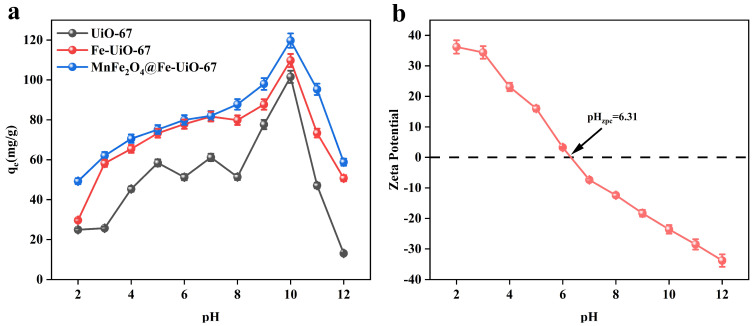
Effect of pH on three adsorbents (**a**); zeta potential of MnFe_2_O_4_@Fe-UiO-67 (**b**).

As shown in Figure 3a, the adsorbent, MnFe_2_O_4_@Fe-UiO-67, showed the best adsorption effect at pH 10. The adsorption was increasing the whole time when the pH increased from 2 to 10, and decreased at pH > 10. To further investigate the adsorption mechanism, the zeta potential of MnFe_2_O_4_@Fe-UiO-67 was detected (Figure 3b). The zeta potential of pHzpc = 6.31; when the pH was <6.31, the surface of the adsorbent Was positively charged, which attracted the negatively charged arsenic ions and had a strong adsorption capacity, and when the pH was >6.31, the surface of the adsorbent was negatively charged, which repelled the arsenic ions and hassled to it having a weakened adsorption capacity. However, the adsorption capacity of the adsorbent was the strongest at pH = 10, indicating that electrostatic adsorption was not the main adsorption mechanism.

### 3.3. Exploration of Arsenic Removal Rate and Adsorption Capacity

**Figure 4:**
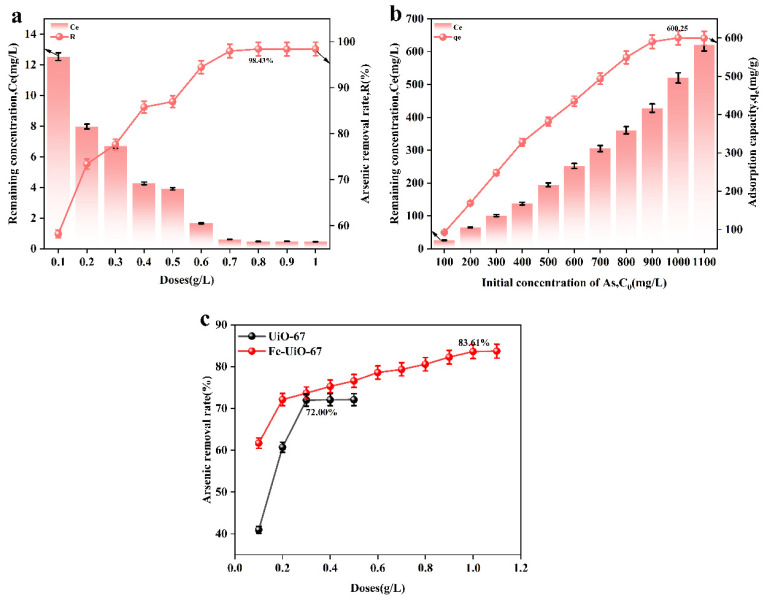
Effect of different dosages of adsorbent (**a**,**c**) and different concentrations of solution on adsorption performance (**b**).

Figure 4a,c show the effect of the adsorbent dosage on adsorbent performance. From Figure 4a, it can be seen that when the adsorbent dosage was 0.8 g/L, the adsorption rate reached a maximum of 98.43%, which is higher than that of UiO-67 and Fe-UiO-67 (Figure 4c). Figure 4b shows the effect of the initial arsenic concentration on adsorbent performance. Prepare an arsenic standard solution with concentrations ranging from 100 to 1000 mg/L using ICP-OES. Transfer 50 mL of the solution into an Erlenmeyer flask, and add the adsorbent at a dosage of 0.8 g/L. When the arsenic solution concentration reached 1000 mg/L, the adsorption capacity of MnFe_2_O_4_@Fe-UiO-67 reached the equilibrium, and the maximum adsorption was 600.25 mg/g, which is higher than that of the other adsorbents reported in the literature (shown in Table 3).

### 3.4. Exploration of Adsorption Kinetics, Isotherms and Thermodynamic

**Figure 5:**
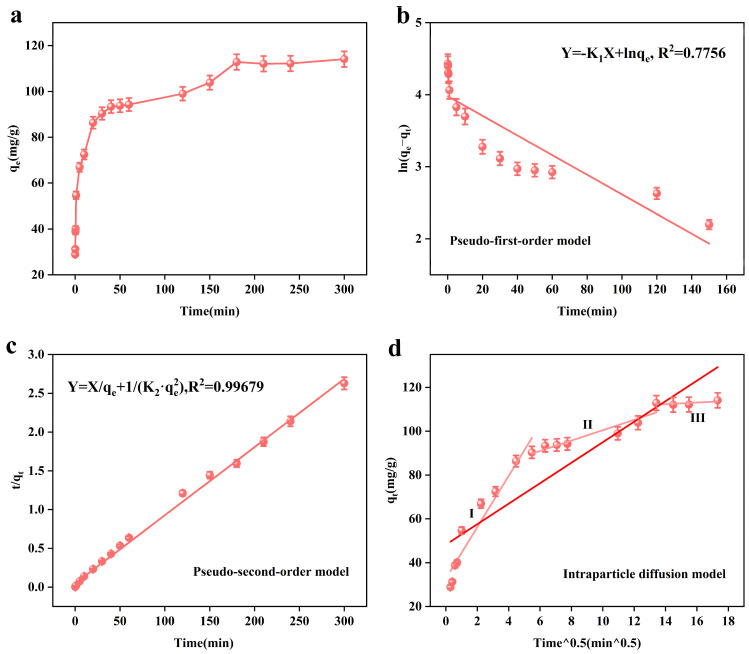
Effect of time on the adsorption performance of the adsorbent, MnFe_2_O_4_@Fe-UiO-67 (**a**); adsorption kinetic pseudo-first-order model and (**b**) pseudo-second-order model (**c**) Intraparticle diffusion model (**d**).

Figure 5a shows the relationship between the adsorption time (t) and adsorption amount (qt). From the figure, we can see that the presence of the adsorbent on the arsenic ions leads to rapid adsorption in the first 40 min, and then the adsorption amount slowly increases until the adsorption equilibrium is reached, after 3 h. This is because at the beginning of the reaction, the adsorbent’s active adsorption sites are more able to quickly bind with the arsenic ions in the solution; after that, the arsenic ions in solution and the adsorption sites of the adsorbent are reduced, resulting in the adsorption rate slowing down and the equilibrium being reached.

Figure 5b–d show the kinetic models fitted by us according to the experimental data. Figure 5b,c show the PFO and PSO, respectively. Table 4 and Table 5 show the relevant kinetic model parameters. From Table 4, it can be seen that the pseudo-second-order kinetic model R^2^ = 0.99679 is larger than the pseudo-first-order kinetic model R^2^ = 0.7756, so the pseudo-second-order kinetic model can better describe the adsorption process of the adsorbent, which is chemisorption. Figure 5d shows the intraparticle diffusion model, and the related parameters are shown in Table 5. In the first stage, the abundance of arsenic ions and the number of adsorption sites lead to the rapid diffusion of arsenic ions; in the second stage, the number of adsorption active sites are reduced due to occupation by arsenic ions, and the amount of arsenic ions is also reduced, so the adsorption rate slows down until it reaches the equilibrium (the third stage).

**Figure 6:**
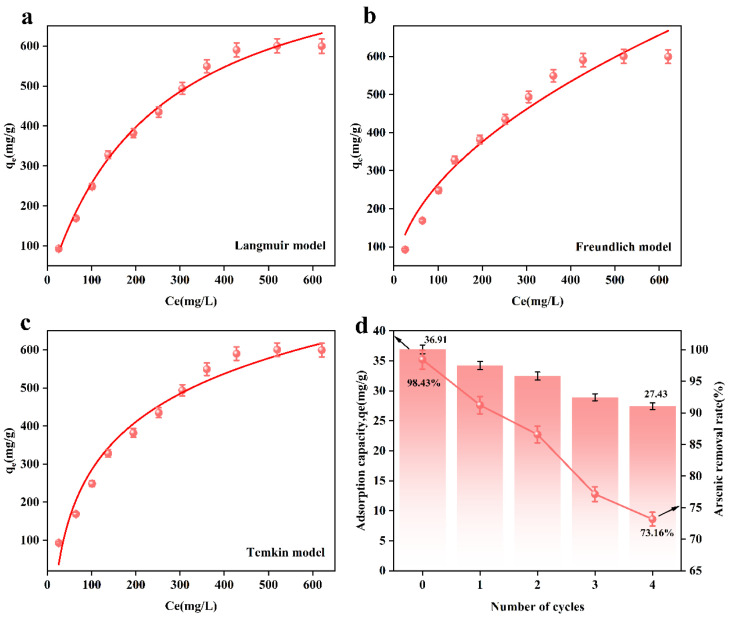
Adsorption isotherm model for MnFe_2_O_4_@Fe-UiO-67; (**a**) Langmuir, (**b**) Freundlich, (**c**) Temkin and adsorption–resolution cycle experiments (**d**).

Figure 6a–c show the adsorption isotherm models fitted based on the experimental data. Table 6 shows the isotherm model parameters of the adsorbent. According to Figure 6 and Table 6, the correlation coefficient, R^2^ = 0.98987, of the Langmuir model of the adsorbent is greater than that of the Freundlich model (R^2^ = 0.95971) and Temkin model (R^2^ = 0.96847). This result indicates that the adsorption process is more consistent with the Langmuir model, i.e., the Langmuir model better describes the adsorption behavior of arsenic ions on the adsorbent, indicating that the form of adsorption tends to be monolayer adsorption.

**Figure 7:**
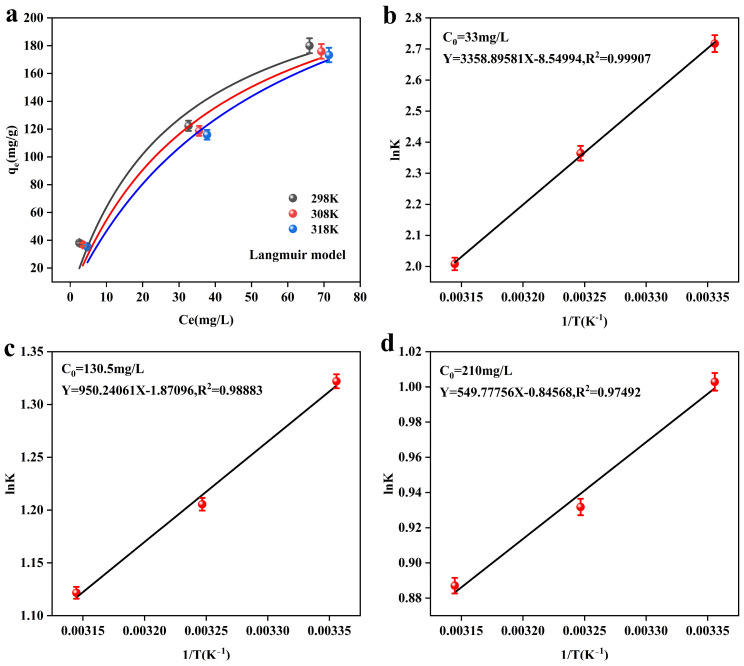
The Langmuir isotherm of the adsorbent at different temperatures (**a**), and Van’t Hoff curves for different initial arsenic concentrations (**b**–**d**).

Figure 7 shows the thermodynamic fitting results of the adsorbent, MnFe_2_O_4_@Fe-UiO-67, and the corresponding thermodynamic parameters are listed in Table 7. Figure 7a shows the Langmuir model of the adsorbent at different temperatures, which shows a decreasing trend in the adsorption capacity (q_e_) with increasing reaction temperatures (298 K–318 K), indicating that the adsorbent has the best adsorption performance at room temperature (298 K). Figure 7a–c are the Van’t Hoff curves of MnFe_2_O_4_@Fe-UiO-67, and the data fitting shows a good result. From the thermodynamic parameters in Table 7, it can be seen that ΔG^0^ < 0, indicating that the adsorption reaction is spontaneous and that ΔG^0^ increases with increasing reaction temperature at the same initial concentration, indicating that a proper decrease in temperature is favorable for the reaction. ΔH^0^ < 0 indicates that the adsorption reaction is exothermic, and lowering the temperature is favorable for the reaction. ΔS^0^ represents the degree of disorder of the reaction system; ΔS^0^ < 0 indicates that the disorder in the adsorption process is reduced, which may be due to the fact that arsenic ions are adsorbed on the MnFe_2_O_4_@Fe-UiO-67 surface. In conclusion, the thermodynamic results indicate that the adsorption process is spontaneous and exothermic and tends to occur at room temperature or lower.

### 3.5. Cyclic Regeneration Capacity of Adsorbent

In order to evaluate the regenerative capacity of the adsorbent, we conducted an adsorption–resolution cycle experiment. The adsorbed MnFe_2_O_4_@Fe-UiO-67 was added to 1 M NaOH solution for arsenic removal. After 12 h, and after arsenic removal, the adsorbent was washed with deionized water until it was neutral. The obtained adsorbent was dried in a desiccator for 2 h, and then the adsorption experiment was performed again. After that, the above operation was repeated.

From Figure 6d, it can be observed that after 4 cycles, the arsenic removal efficiency of MnFe_2_O_4_@Fe-UiO-67 decreased from 98.43% to 73.16%, and the adsorption capacity decreased from 36.91 mg/g to 27.43 mg/g. This may have been due to incomplete desorption, where a small amount of As ions remained in the pores of the adsorbent, affecting its adsorption efficiency. Nevertheless, even after four cycles, the adsorbent still maintained a relatively high arsenic removal efficiency, indicating its excellent regeneration capability and long-term application potential.

## 4. Reaction Mechanisms

**Figure 8:**
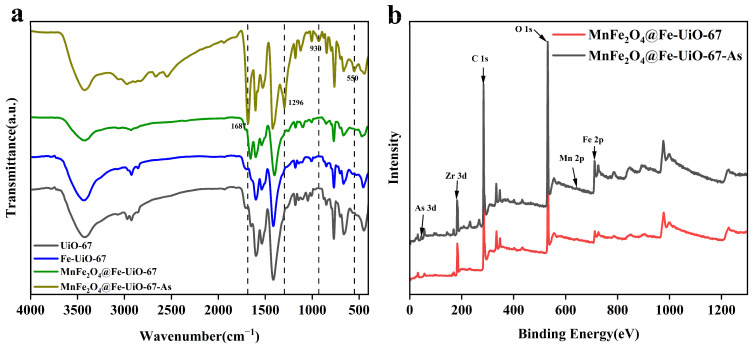
FT-IR (**a**) and XPS gross spectra of adsorbents (**b**).

As shown in Figure 8a, the IR spectra of UiO-67 and Fe-UiO-67 are the same, indicating that they are structurally the same. The four extra bonds after the adsorption of MnFe_2_O_4_@Fe-UiO-67 are Fe-O bonds (550 cm^−1^) [34]; the peaks at 930 cm^−1^ correspond to the As-O bond [46], indicating that the active sites on the adsorbent form coordination bonds with As ions, thereby enabling the removal of arsenic. The peaks observed at 1296 cm⁻¹ and 1687 cm⁻¹ may emerge due to structural modifications induced by the adsorption process. Figure 8b shows the total XPS spectra before and after adsorption, in which the content of the element Fe increased after adsorption and As appeared, indicating that the structure of MnFe_2_O_4_@Fe-UiO-67 changed after adsorption and arsenic ions were adsorbed on it, and that there was no obvious peak for Mn due to the trace amounts of it.

**Figure 9:**
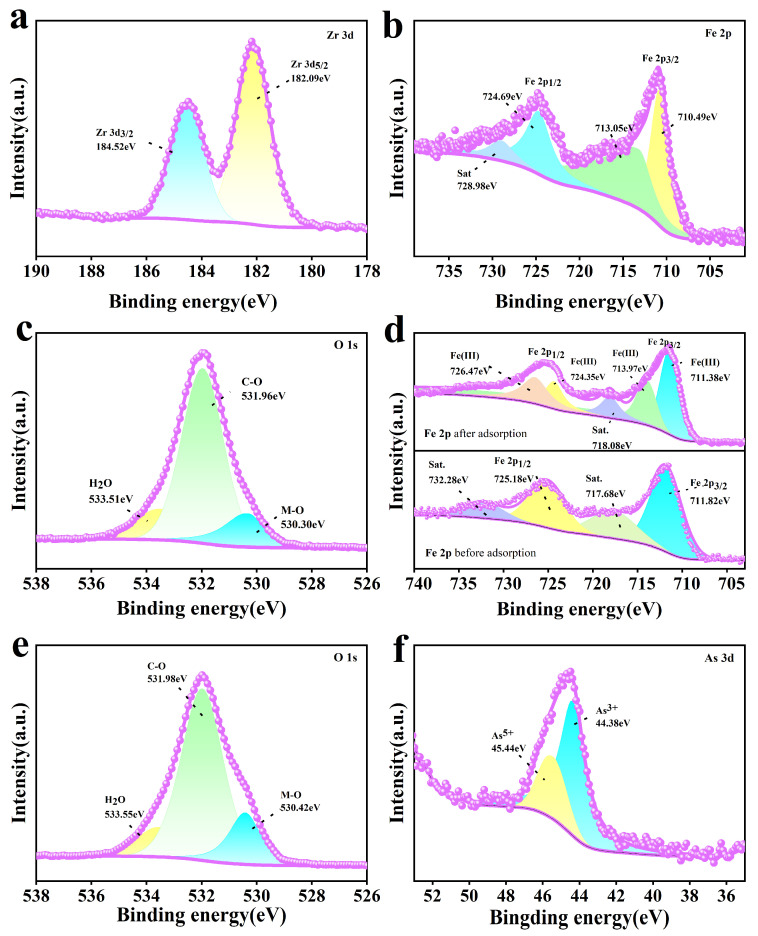
XPS of adsorbents Fe-UiO-67 and MnFe_2_O_4_@Fe-UiO-67. O1s and Fe2p (**a**,**b**) of Fe-UiO-67, and O 1s of MnFe_2_O_4_@Fe-UiO-67 (**c**); Fe 2p (**d**) before and after arsenic adsorption, and O 1s (**e**) and As 3d (**f**) after arsenic adsorption of MnFe_2_O_4_@Fe-UiO-67.

Figure 9 shows the XPS spectra of the adsorbent. The Zr 3d and Fe 2p spectra of Fe-UiO-67 are shown in Figure 9a and b, respectively. In the Zr 3d spectrum, two characteristic peaks are shown at 182.09 eV and 184.52 eV, attributed to Zr 3d_5/2_ and Zr 3d_3/2_, respectively [47,48]. In the Fe 2p spectra, the characteristic peaks at 710.49 eV and 724.69 eV are attributed to Fe 2p_3/2_ and Fe 2p_1/2_, and the appearance of the characteristic peaks at 713.05 eV and 728.98 eV can be assigned to the satellite peaks, confirming the successful doping of Fe^3+^ on UiO-67 [49,50]. According to the O 1s spectra of MnFe_2_O_4_@Fe-UiO-67 before and after adsorption (Figure 9c,e), the peak area of the M–O bond after adsorption was significantly increased, which, in combination with the FT-IR spectra (Figure 8a), indicates the generation of an As–O bond and Fe–O bond. Figure 9d shows the Fe 2p mapping; the peaks at 711.82 eV and 725.81 eV before adsorption can be attributed to Fe 2p_3/2_ and Fe 2p_1/2_, while 717.68 eV and 732.28 eV are satellite peaks [49]. After adsorption, the characteristic peaks of the Fe 2p spectra were at 711.38 eV/713.97 eV and 724.35 eV/726.47 eV, and the satellite peaks were 718.08 eV and 726.47 eV. The peaks were significantly changed compared to those before adsorption, which indicates that a chemical reaction occurred in the system. Figure 9f shows the As 3D spectrum with peaks at 44.38 eV and 45.44 eV for As^3+^ and As^5+^ respectively [51]. It indicates that arsenic ions were adsorbed onto the MnFe_2_O_4_@Fe-UiO-67.

**Figure 10:**
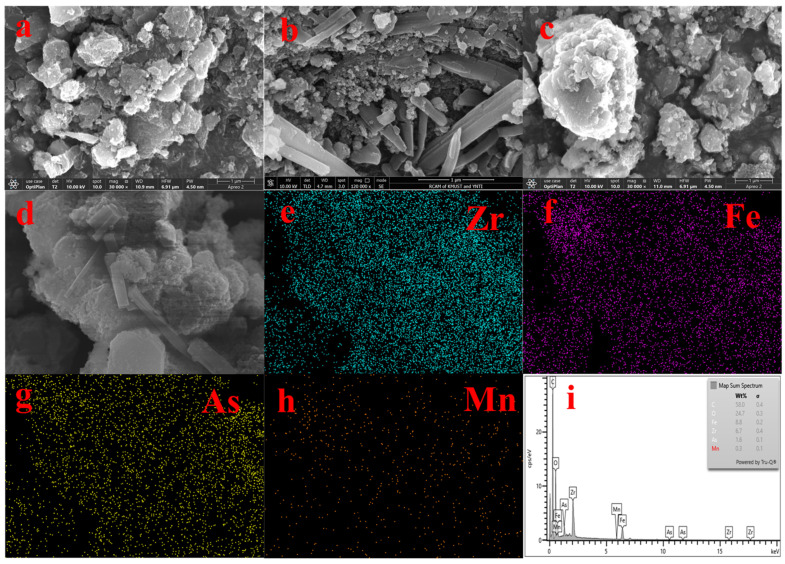
SEM (**a**–**d**) and EDS (**e**–**i**) of MnFe_2_O_4_@Fe-UiO-67 after adsorption.

Figure 10 shows the SEM plots (a, b, c, d) of MnFe_2_O_4_@Fe-UiO-67 after adsorption. By comparing the morphologies before and after adsorption (Figure 2d,e show the morphology before adsorption), slight changes occurred in the structure of the adsorbent, which confirms the conclusions drawn from Figure 7a,b. The appearance of arsenic elements in the EDS plots (e, f, h, i) suggests that arsenic ions are adsorbed on it.

## 5. Environmental Applications Outlook

The adsorbent is synthesized with the help of a low-temperature energy-saving process using green materials as raw materials, which are not only environmentally friendly but also economically feasible. The adsorbent can be recycled many times, and thermodynamic analysis shows that the adsorbent can achieve high adsorption efficiency at room temperature without additional energy consumption, which reduces energy costs. The core component of the adsorbent is Zr-MOF with a fcu topology, which can be introduced into the defective sites by modulators to form more open Zr sites (Lewis acid sites), resulting in superior adsorption performance [52,53]. Meanwhile, both Zr-MOF and MnFe₂O₄ exhibit excellent adsorption performance toward various heavy metal ions (such as Pb^2+^, Cd^2+^, Cu^2+^, etc.) and organic pollutants, which endows them with a broader application scope [54,55,56].

Currently, as a leader in the industry, BASF has taken the lead in overcoming technical challenges and successfully established a commercial production chain for MOF materials [57]. With an annual production capacity of hundreds of tons, it has laid a solid material foundation for the in-depth application and rapid popularization of MOF materials in the field of technology. With the continuous progress of materials science, on the one hand, through the refined improvement of the synthesis process and the exploration of the potential for large-scale production, the preparation cost of MOFs materials has been significantly reduced. This not only broadens the application market and endows the materials with broad application prospects, but also remarkably enhances the cost–performance ratio of MOF adsorbents, making their promotion and application in small and medium-sized enterprises more smooth. On the other hand, these measures have promoted the integration of MOF materials into multiple industries such as environmental protection, chemical engineering, energy, biomedicine, and electronics, helping to solve practical problems. This further highlights the huge development potential of MOF materials and paves the way for a broader future for them.

## 6. Conclusions

In this study, a simple and green synthesis method was used to synthesize the new composite nanomaterial MnFe_2_O_4_@Fe-UiO-67. The material showed excellent adsorption performance, with an arsenic removal rate of 98.43% and an adsorption capacity of 600.25 mg/g. Characterization analyses by XRD and SEM proved that MnFe_2_O_4_ and Fe ions were successfully loaded on UiO-67. The experimental results showed that the process of arsenic adsorption on MnFe_2_O_4_@Fe-UiO-67 conformed to the Langmuir and pseudo-second-order kinetic models, indicating that the adsorption process was monomolecular-layer chemisorption. Through thermodynamic analysis, we were able to confirm that this adsorption reaction is a spontaneous exothermic reaction, and it exhibits excellent adsorption performance at room temperature without the need for additional energy consumption. Moreover, after four adsorption–desorption cycles, the adsorbent still exhibited high adsorption performance, demonstrating its excellent stability and regeneration capability, which is of significant application value in practical water treatment.

## Figures and Tables

**Table 1:** Elemental composition analysis of diluted arsenic-containing wastewater (mg/L).

Elements	As	Pb	Cu	Ni	Cr	Cd
Content (mg/g)	22.21	-	0.3945	0.1571	-	0.1565

**Table 2:** Data related to N_2_ adsorption–desorption of adsorbents.

Absorbents	Langmuir Surface Area (m^2^g^−1^)	Pore Volume (cm^3^g^−1^)	Pore Size (nm)
UiO-67	1789.7537	0.499563	2.0109
Fe-UiO-67	2282.5873	0.644241	2.6982
MnFe_2_O_4_@Fe-UiO-67	2522.0055	0.622448	3.6110

**Table 3:** Adsorption capacity of various adsorbents for arsenic ions in the literature.

Arsenic Species	Adsorbents	Surface Area (m^2^/g)	Pore Volume (cm^3^/g)	pH	*q_m_* (mg/g)	Reference
As(III) and As(V)	MnFe_2_O_4_@Fe-UiO-67	2522.0055	0.622448	10	600.25	this work
As(III) and As(V)	NF/MIL-101 (Cr)	-	-	4	132.615	[41]
As(V)	MIL-53 (Fe)	14	0.012	5	21.27	[42]
ASA	UiO-67-NH_2_	750	0.8	4	178	[43]
As(V)	Fe/Mn-MOF	-	-	11	228.79	[44]
As(III)	Fe_3_O_4_/CSAC	575.607	0.678	7	80.99	[45]

ASA: organic arsenic; CSAC: cigarette soot-activated carbon.

**Table 4:** Kinetic model parameters associated with MnFe_2_O_4_@Fe-UiO-67.

	Parameters	Value
Pseudo-first-order model	K	0.01365
	R^2^	0.7756
Pseudo-second-order model	K	0.0088
	R^2^	0.99679

**Table 5:** Intraparticle diffusion model parameters.

Parameters	Part I	Part II	Part III
K	11.70314	2.36325	0.3655
R^2^	0.92886	0.89186	0.42337

**Table 6:** Adsorption isotherm model parameters.

Adsorbent	Langmuir		Freundlich		Temkin	
	K_L_	R^2^	K_F_	R^2^	K_T_	R^2^
MnFe_2_O_4_@Fe-UiO-67	0.00412	0.98987	25.5006	0.95971	0.04762	0.96847

**Table 7:** Thermodynamic parameters of As adsorption by MnFe_2_O_4_@Fe-UiO-67.

Concentration (mg/L)	Temperature (K)	K	LnK	ΔG^0^ (KJ/mol)	ΔH^0^ (KJ/mol)	ΔS^0^ (J/mol/K)
33	298	15.13856	2.717245	−6.73217	−27.9259	−71.0842
	308	10.63761	2.364396	−6.05454		
	318	7.448861	2.008061	−5.30902		
130.5	298	3.750766	1.32196	−3.27525	−7.9003	−15.5552
	308	3.338608	1.205554	−3.08708		
	318	3.070048	1.121693	−2.96559		
210	298	2.726068	1.00286	−2.4846	−4.57085	−7.03098
	308	2.538972	0.931759	−2.3859		
	318	2.428016	0.887074	−2.3452		

## Data Availability

Data will be made available on request.
